# Supplementary material for: Clinical Outcomes and Cost Analysis in Patients with Heart Failure Undergoing Transcatheter Edge-to-Edge Repair for Mitral Valve Regurgitation
Source: J Pers Med. 2024 Sep 15;14(9):978. doi: 10.3390/jpm14090978 (PMC11433586; doi:10.3390/jpm14090978)
Supplement: Supplementary file 1 [file jpm-14-00978-s001.zip › jpm-3106717-supplementary.pdf]

Supplement Material

Methods section:

Propensity Score development

We created three separate propensity scores to use as covariates in our regression models. We used a multivariable logistic regression with systolic vs diastolic, systolic vs mixed, and diastolic vs mixed as the outcome variables. We chose preoperative variables that differed between the groups and removed any that caused standard errors to be large. Variables included in the multivariable logistic regression model for propensity-score development included the following:

1. Systolic vs Diastolic: age, gender, NYHA, CDK, dyslipidemia, PVD, diabetes, immunocompromised, cancer within 5 years, prior cardiovascular intervention, previous PCI, prior valve surgery, prior MI, prior stroke, cardiogenic shock, preop a-fib, previous AICD, prior history of arrhythmias, number of diseased vessels, mitral regurgitation primary mechanism
2. Systolic vs Mixed: NYHA, obesity, preop creatinine levels, COPD, CKD, home O2, dyslipidemia, CBVD, PVD, cancer within 5 years, mediastinal radiation, prior cardiovascular intervention, prior MI, prior stroke, number of diseased vessels, mitral regurgitation primary mechanism
3. Diastolic vs Mixed: age, gender, race, NYHA, BMI, preop creatinine, CKD, diabetes, mediastinal radiation, prior cardiovascular intervention, previous PCI, prior valve surgery, preop a-fib, prior history of arrhythmias, previous AICD, pacemaker, preop EF, number of diseased vessels

In the Table 2 analysis, we added the propensity score for the appropriate comparison to a linear or logistic regression model with the comparison group and propensity score as covariates. Example:

Time in OR = $\beta 1 + \beta 2$(systolic (0) vs diastolic (1)) + $\beta 3$(p-score for systolic vs diastolic)

Table 6 added the propensity scores to the Cox regression and Fines and Grays regression analyses.

Multivariable models

In Table 6 we created multivariable models as a sensitivity analysis to the propensity-adjusted models. We ran univariable regressions for each outcome and comparator group. Then we ran multivariable regressions for each outcome with the comparator group (ex. Systolic vs diastolic) and one preoperative variable. Any preoperative variables that changed the hazard ratio of the comparator group by more than 10% were included in the larger multivariable model. Postoperative MR was also tested as a possible confounder and included in the models if it was deemed a confounder.

Table section

Table S1: Univariate, propensity-adjusted, and multivariable analysis for long-term outcomes.

Note: HR: hazard ratio, SHR: sub-distribution hazard ratio (competing risk).

	Univariable	Propensity Adjusted(a)	Multivariable (b)
**All-Cause Mortality**	HR (95% CI)	HR (95% CI)	HR (95% CI)
Diastolic (Ref Systolic)	0.5 (0.3, 0.9)*	0.6 (0.3, 1.3)	0.5 (0.2, 1.1)
Mixed (Ref Systolic)	1.2 (0.6, 2.2)	0.96 (0.5, 2.0)	1.2 (0.5, 2.7)
Mixed (Ref Diastolic)	2.4 (1.4, 4.2)**	1.3 (0.6, 2.9)	1.2 (0.6, 2.6)
**Cardiac Death**	SHR (95% CI)	SHR (95% CI)	SHR (95% CI)
Diastolic (Ref Systolic)	0.8 (0.3, 2.0)	1.1 (0.4, 3.4)	1.1 (0.4, 2.8)
Mixed (Ref Systolic)	1.4 (0.6, 3.5)	1.1 (0.4, 3.2)	0.7 (0.2, 2.2)
Mixed (Ref Diastolic)	1.7 (0.8, 3.7)	1.2 (0.3, 4.5)	0.8 (0.2, 3.2)
**MACCE**	HR (95% CI)	HR (95% CI)	HR (95% CI)
Diastolic (Ref Systolic)	0.5 (0.3, 0.91)*	0.6 (0.3, 1.2)	0.6 (0.3, 1.1)
Mixed (Ref Systolic)	1.2 (0.7, 2.3)	1.1 (0.5, 2.2)	1.3 (0.6, 2.8)
Mixed (Ref Diastolic)	2.3 (1.4, 3.7)**	1.4 (0.7, 3.0)	1.5 (0.7, 3.2)
**Stroke**	SHR (95% CI)	SHR (95% CI)	SHR (95% CI)
Diastolic (Ref Systolic)	1.7 (0.4, 7.3)	0.99 (0.2, 4.3)	0.8 (0.1, 4.6)
Mixed (Ref Systolic)	1.4 (0.2, 7.8)	0.9 (0.1, 9.8)	1.4 (0.1, 25.1)
Mixed (Ref Diastolic)	0.8 (0.2, 3.2)	0.7 (0.2, 3.1)	0.7 (0.1, 5.0)
**Myocardial Infarction**	SHR (95% CI)	SHR (95% CI)	SHR (95% CI)
Diastolic (Ref Systolic)	0.4 (0.1, 2.6)	0.1 (0.01, 3.2)	0.6 (0.1, 4.1)
Mixed (Ref Systolic)	0.9 (0.1, 6.4)	0.8 (.02, 28.8)	1.1 (0.2, 5.1)
Mixed (Ref Diastolic)	2.5 (0.3, 19.9)	13.6 (1.6, 113)*	20.9 (3.6, 120)**
**Repeat Intervention**	SHR (95% CI)	SHR (95% CI)	SHR (95% CI)
Diastolic (Ref Systolic)	1.6 (0.4, 7.2)	1.2 (0.4, 4.3)	0.9 (0.3, 3.0)
Mixed (Ref Systolic)	0.9 (0.1, 6.7)	1.1 (0.04, 29)	1.3 (0.3, 6.1)
Mixed (Ref Diastolic)	0.6 (0.1, 2.8)	0.6 (0.2, 1.6)	1.4 (0.4, 5.1)
**New Pacemaker Implantation**	SHR (95% CI)	SHR (95% CI)	SHR (95% CI)

Diastolic (Ref Systolic)	NA	NA	NA
Mixed (Ref Systolic)	NA	NA	NA
Mixed (Ref Diastolic)	8.3 (2.2, 30.9)	6.5 (1.7, 25.1)**	9.1 (3.1, 26.3)***
*** <0.001			
**<0.01			
*<0.05			
a. propensity adjusted also includes postop MR			
b. postoperative MR tested as confounder, included if it changed univariable results by 10%			
